# Anti-periodic oscillations of bidirectional associative memory (BAM) neural networks with leakage delays

**DOI:** 10.1186/s13660-018-1658-2

**Published:** 2018-04-03

**Authors:** Changjin Xu, Lilin Chen, Ting Guo

**Affiliations:** 1 Guizhou Key Laboratory of Economics System Simulation, Guizhou University of Finance and Economics, Guiyang, P.R. China; 2 School of Mathematics and Statistics, Guizhou University of Finance and Economics, Guiyang, P.R. China; 3 School of Mathematics and Statistics, Central South University, Changsha, P.R. China

**Keywords:** 34C25, 34K13, 34K25, BAM neural networks, Anti-periodic solution, Exponential stability, Time-varying delay, Leakage term

## Abstract

In this article, we discuss anti-periodic oscillations of BAM neural networks with leakage delays. A sufficient criterion guaranteeing the existence and exponential stability of the involved model is presented by utilizing mathematic analysis methods and Lyapunov ideas. The theoretical results of this article are novel and are a key supplement to some earlier studies.

## Introduction

In the past several decades, the dynamics of BAM neural networks has been widely investigated for their essential applications in classification, pattern recognition, optimization, signal and image processing, and so on [1–41]. In 1987, Kosko [42] proposed the following BAM neural network:

1.1 $$\begin{aligned} \textstyle\begin{cases} \frac{du_{i}(t)}{dt}=-a_{i}u_{i}(t)+\sum_{j=1}^{n}a_{ij}f_{j}(v_{j}(t-\sigma _{j}(t)))+I_{i},\\ \frac{dv_{i}(t)}{dt}=-b_{i}v_{i}(t)+\sum_{j=1}^{n}b_{ij}g_{j}(u_{j}(t-\tau_{j}(t)))+J_{i}, \end{cases}\displaystyle \end{aligned}$$

where $i=1,2,\ldots,n,t>0$. Here, $a_{i}>0,b_{i}>0$ denote the time scales of the respective layers of the network; $-a_{i}u_{i}(t)$ and $-b_{i}v_{i}(t)$ stand for the stabilizing negative feedback of the model. Noticing that the leakage delay often appears in the negative feedback term of neural networks (see [43–47]), Gopalsmay [48] studied the stability of the equilibrium and periodic solutions for the following BAM neural network:

1.2 $$\begin{aligned} \textstyle\begin{cases} \frac{dx_{i}(t)}{dt}=-a_{i}x_{i}(t-\tau_{i}^{(i)})+\sum_{j=1}^{n}a_{ij}f_{j}(y_{j}(t-\sigma_{j}^{(2)}))+I_{i},\\ \frac{dy_{i}(t)}{dt}=-b_{i}y_{i}(t-\tau_{i}^{(2)})+\sum_{j=1}^{n}b_{ij}g_{j}(x_{j}(t-\sigma_{j}^{(1)}))+J_{i}, \end{cases}\displaystyle \end{aligned}$$

where $i=1,2,\ldots,n,t>0$. Since the delays in neural networks are usually time-varying in the real world, Liu [49] discussed the global exponential stability for the following general BAM neural network with time-varying leakage delays:

1.3 $$\begin{aligned} \textstyle\begin{cases} \frac{dx_{i}(t)}{dt}=-a_{i}x_{i}(t-\delta_{i}(t))+\sum_{j=1}^{n}a_{ij}f_{j}(y_{j}(t-\sigma_{ij}(t))+I_{i},\\ \frac{dy_{i}(t)}{dt}=-b_{i}y_{i}(t-\eta_{i}(t)+\sum_{j=1}^{n}b_{ij}g_{j}(x_{j}(t-\tau _{ij}(t))+J_{i}. \end{cases}\displaystyle \end{aligned}$$

However, so far, there have been rare reports on the existence and exponential stability of anti-periodic solutions of neural networks, especially for neural networks with leakage delays. Furthermore, the existence of anti-periodic solutions can be applied to help better describe the dynamical properties of nonlinear systems [49–65]. So we think that the investigation on the existence and stability of anti-periodic solutions for neural networks with leakage delays has significant value. Inspired by the ideas and considering the change of system parameters in time, we can modify neural network model (1.3) as follows:

1.4 $$\begin{aligned} \textstyle\begin{cases} \frac{dx_{i}(t)}{dt}=-a_{i}x_{i}(t-\delta_{i}(t))+\sum_{j=1}^{n}a_{ij}(t)f_{j}(y_{j}(t-\sigma_{ij}(t)))+I_{i}(t),\\ \frac{dy_{i}(t)}{dt}=-b_{i}y_{i}(t-\eta_{i}(t))+\sum_{j=1}^{n}b_{ij}(t)g_{j}(x_{j}(t-\tau_{ij}(t)))+J_{i}(t). \end{cases}\displaystyle \end{aligned}$$

The main objective of this article is to analyze the exponential stability behavior of anti-periodic oscillations of (1.4). Based on the fundamental solution matrix, Lyapunov function, and fundamental function sequences, we establish a sufficient condition ensuring the existence and global exponential stability of anti-periodic solutions of (1.4). The derived findings can be used directly to numerous specific networks. Besides, computer simulations are performed to support the obtained predictions. Our findings are a good complement to the work of Gopalsmay [48] and Liu [49].

The paper is planned as follows. In Sect. 2, several notations and preliminary results are prepared. In Sect. 3, we give a sufficient condition for the existence and global exponential stability of anti-periodic solution of (1.4). In Sect. 4, we present an example to show the correctness of the obtained analytic findings.

### Remark 1.1

A time delay that exists in the negative feedback term (or called leakage term or forgetting term) of neural networks is called leakage delay. If there exists an anti-periodic solution in a dynamical system, then we can say that the system has anti-periodic oscillations.

## Preliminary results

In this segment, several notations and lemmas will be given.

For any vector $V=(v_{1},v_{2},\dots,v_{n})^{T}$ and matrix $D=(d_{ij})_{n\times{n}}$, we define the norm as

$$\Vert V \Vert = \Biggl(\sum_{i=1}^{n}v_{i}^{2} \Biggr)^{\frac{1}{2}},\qquad \Vert D \Vert = \Biggl(\sum _{i=1}^{n}d_{ij}^{2} \Biggr)^{\frac{1}{2}}, $$

respectively. Let

$$\begin{aligned} &\tau=\Bigl\{ \sup_{t\in{R}}\max_{1\leq{i}\leq{n}} \delta_{i}(t),\sup_{t\in {R}}\max_{1\leq{i}\leq{n}} \eta_{i}(t), \sup_{t\in{R}}\max_{1\leq{i,j}\leq {n}} \sigma_{ij}(t),\sup_{t\in{R}}\max_{1\leq{i,j}\leq{n}} \tau_{ij}(t)\Bigr\} , \\ &\varphi(s)=\bigl(\varphi_{1}(s),\varphi_{2}(s),\ldots, \varphi_{n}(s)\bigr)^{T},\qquad \psi(s)=\bigl(\psi_{1}(s), \psi_{2}(s),\ldots,\psi_{n}(s)\bigr)^{T}, \end{aligned}$$

where $\varphi_{i}(s)\in{C}([-\tau,0],R), \psi_{i}(s)\in{C}([-\tau,0],R),i=1,2,\ldots,n$, we define

$$\Vert \varphi \Vert =\sup_{-\tau\leq{s}\leq0} \Biggl(\sum _{i=1}^{n} \bigl\vert \varphi _{i}(s) \bigr\vert ^{2} \Biggr)^{\frac{1}{2}}, \qquad \Vert \psi \Vert =\sup _{-\tau\leq{s}\leq0} \Biggl(\sum_{i=1}^{n} \bigl\vert \psi_{i}(s) \bigr\vert ^{2} \Biggr)^{\frac{1}{2}}. $$

We assume that system (1.4) always satisfies the following initial conditions:

2.1 $$\begin{aligned} \textstyle\begin{cases} x_{i0}=\varphi_{i}(s),& s\in[-\tau,0],\\ y_{i0}=\psi_{i}(s),& s\in[-\tau,0]. \end{cases}\displaystyle \end{aligned}$$

Let $x(t)=(x_{1}(t),x_{2}(t),\ldots,x_{n}(t))^{T}, y(t)=(y_{1}(t),y_{2}(t),\ldots,y_{n}(t))^{T}$ be the solution of system (1.4) with initial conditions (2.1). We say the solution $x(t)=(x_{1}(t),x_{2}(t),\ldots,x_{n}(t))^{T}, y(t)=(y_{1}(t),y_{2}(t),\ldots,y_{n}(t))^{T}$ is *T*-anti-periodic on $R^{2n}$ if $x_{i}(t+T)=-x_{i}(t), y_{i}(t+T)=-y_{i}(t)$ for all $t\in{R}$ and $i=1,2,\dots,n$, where *T* is a positive constant.

Throughout this paper, for $i,j=1,2,\ldots,n$, it will be assumed that there exist constants such that

$$\begin{aligned} &\delta_{i}^{+}=\sup_{t\in{R}} \delta_{i}(t), \qquad \eta_{i}^{+}=\sup _{t\in{R}}\eta_{i}(t),\qquad a_{ij}^{+}= \sup_{t\in{R}}a_{ij}(t), \\ & b_{ij}^{+}= \sup_{t\in{R}}b_{ij}(t),\qquad \tau_{ij}^{+}= \sup_{t\in{R}}\tau_{ij}(t), \qquad \sigma_{ij}^{+}= \sup_{t\in{R}}\sigma_{ij}(t) \end{aligned}$$

and $t-\delta_{i}(t)>0, t-\eta_{i}(t)>0$.

We also assume that the following conditions hold.

(H1) For $j=1,2,\ldots,n$, there exist constants $L_{jf}>0, L_{jg}>0, M_{j}^{f}>0$, and $M_{j}^{g}>0$ such that

$$\textstyle\begin{cases} \vert f_{j}(u )-f_{j}(v ) \vert \leq L_{jf} \vert u -v \vert , \quad \vert f_{j}(u) \vert < M_{j}^{f},\\ \vert g_{j}(u )-g_{j}(v ) \vert \leq L_{jg} \vert u -v \vert ,\quad \vert g_{j}(u) \vert < M_{j}^{g}, \end{cases} $$

for all $u, v \in{R}$.

(H2) For all $t,u\in{R}$ and $i,j=1,2,\ldots,n$,

$$\textstyle\begin{cases} a_{ij}(t+T)f_{j}(u)=-a_{ij}(t)f_{j}(-u),\qquad b_{ij}(t+T)g_{j}(u)=-b_{ij}(t)g_{j}(-u),\\ \delta_{i}(t+T)=\delta_{i}(t),\qquad \eta_{i}(t+T)=\eta_{i}(t),\qquad \sigma_{ij}(t+T)=\sigma_{ij}(t),\\ \tau_{ij}(t+T)=\tau_{ij}(t),\qquad I_{i}(t+T)=-I_{i}(t),\qquad J_{i}(t+T)=-J_{i}(t), \end{cases} $$

where *T* is a positive constant.

It is clear that the conditions can be fulfilled; for example, let $a_{ij}(t)=0.2 \vert \cos t \vert , f_{j}(u)=u^{2j-1},i,j=1,2,\ldots,n$, then we have $a_{ij}(t+T)f_{j}(u)=-a_{ij}(t)f_{j}(-u)$.

(H3) The following inequality holds:

$$\frac{\sqrt{2}}{\alpha}\bigl(a_{i}\delta_{i}^{+}+b_{i} \eta_{i}^{+}\bigr)< 1, $$

where $\alpha=\min_{1\leq{i}\leq{n}}\{a_{i},b_{i}\}, i=1,2,\dots,n$.

### Definition 2.1

The solution $(x^{*}(t),y^{*}(t))^{T}$ of system (1.4) is said to globally exponentially stable if there exist constants $\beta>0$ and $M>1$ such that

$$\sum_{i=1}^{n} \bigl\vert x_{i}(t)-x_{i}^{*}(t) \bigr\vert ^{2}+\sum _{i=1}^{n} \bigl\vert y_{i}(t)-y_{i}^{*}(t) \bigr\vert ^{2}\leq {M}e^{-\beta t}\bigl( \bigl\Vert \varphi- \varphi^{*} \bigr\Vert ^{2}+ \bigl\Vert \psi-\psi^{*} \bigr\Vert ^{2}\bigr) $$

for each solution $(x(t),y(t))^{T}$ of system (1.4).

Next, we present three important lemmas which are necessary for proving our main results in Sect. 3.

### Lemma 2.1

*Let*

$$A=\left ( \begin{matrix} -a_{i} & 0 \\ 0 & -b_{i} \end{matrix} \right ),\qquad \alpha=\min_{1\leq{i}\leq{n}}\{a_{i},b_{i} \}, $$

*then we have*

$$\Vert \exp A t \Vert \leq\sqrt{2}e^{-\alpha t} $$

*for all* $t\geq0$.

### Proof

Since

$$A=\left ( \begin{matrix} -a_{i} & 0 \\ 0 & -b_{i} \end{matrix} \right ), $$

it follows that

$$\exp A t=\left ( \begin{matrix} e^{-a_{i}t} & 0 \\ 0 & e^{-b_{i}t} \end{matrix} \right ). $$

By the definition of matrix norm, we get

$$\Vert \exp A t \Vert = \bigl(e^{ -2a_{i}t}+e^{ -2b_{i}t} \bigr)^{\frac{1}{2}}\leq\sqrt {2}e^{-\alpha t}. $$

 □

### Lemma 2.2

*Assume that*

$$\mathrm{(H4)} \textstyle\begin{cases} -2a_{i}+a_{i}^{2}\delta_{i}^{+}+\sum_{j=1}^{n}a_{i}\delta _{i}^{+}a_{ij}^{+}L_{jf}^{2\xi_{j}}+\sum_{j=1}^{n}a_{ij}^{+}L_{jf}^{2\xi_{j}}\\ \quad{}+\sum_{i=1}^{n}a_{i}^{2}\delta_{i}^{+}+\sum_{j=1}^{n}b_{i}\eta _{i}^{+}b_{ij}^{+}L_{jg}^{2(1-\varepsilon _{j})}+b_{ij}^{+}L_{jg}^{2(1-\varepsilon_{j})}< 0, \\ {-}2b_{i}+b_{i}^{2}\eta_{i}^{+}+\sum_{j=1}^{n}b_{i}\eta _{i}^{+}b_{ij}^{+}L_{jg}^{2\varepsilon_{j}}+\sum_{j=1}^{n}b_{ij}^{+}L_{jg}^{2\varepsilon_{j}}\\ \quad{} +\sum_{i=1}^{n}a_{i}^{2}\delta_{i}^{+}a_{ij}^{+}L_{jf}^{2(1-\xi _{j})}+a_{ij}^{+}L_{jf}^{2(1-\xi_{j})}+b_{i}\eta_{i}^{+}< 0, \end{cases} $$

*where* $0\leq\varepsilon_{i}\leq1\ (i=1,2,3,4,5,6)$ *are any constants*. *Then there exists* $\beta>0$ *such that*

$$\begin{aligned} & \beta-2a_{i}+a_{i}^{2}\delta_{i}^{+}+ \sum_{j=1}^{n}a_{i}\delta _{i}^{+}a_{ij}^{+}L_{jf}^{2\xi_{j}}+ \sum_{j=1}^{n}a_{ij}^{+}L_{jf}^{2\xi _{j}} \\ & \quad{}+\sum_{i=1}^{n}a_{i}^{2} \delta_{i}^{+}e^{\beta \delta_{i}^{+}}+\sum _{j=1}^{n}b_{i}\eta_{i}^{+}b_{ij}^{+}L_{jg}^{2(1-\varepsilon _{j})}e^{\beta\tau_{ij}^{+}}+b_{ij}^{+}L_{jg}^{2(1-\varepsilon_{j})}e^{\beta \tau_{ij}^{+}} \leq0, \\ & \beta-2b_{i}+b_{i}^{2}\eta_{i}^{+}+ \sum_{j=1}^{n}b_{i}\eta _{i}^{+}b_{ij}^{+}L_{jg}^{2\varepsilon_{j}}+ \sum_{j=1}^{n}b_{ij}^{+}L_{jg}^{2\varepsilon_{j}} \\ & \quad{}+\sum_{i=1}^{n}a_{i}^{2} \delta_{i}^{+}a_{ij}^{+}L_{jf}^{2(1-\xi_{j})}e^{\beta \delta_{ij}^{+}}+a_{ij}^{+}L_{jf}^{2(1-\xi_{j})}e^{\beta\delta _{ij}^{+}}+b_{i} \eta_{i}^{+}e^{\beta\eta_{i}^{+}}\leq0. \end{aligned}$$

### Proof

Let

$$\begin{aligned} \varrho_{1i}(\beta)={}&\beta-2a_{i}+a_{i}^{2} \delta_{i}^{+}+\sum_{j=1}^{n}a_{i} \delta_{i}^{+}a_{ij}^{+}L_{jf}^{2\xi_{j}}+ \sum_{j=1}^{n}a_{ij}^{+}L_{jf}^{2\xi_{j}} \\ & {}+\sum_{i=1}^{n}a_{i}^{2} \delta_{i}^{+}e^{\beta \delta_{i}^{+}}+\sum _{j=1}^{n}b_{i}\eta_{i}^{+}b_{ij}^{+}L_{jg}^{2(1-\varepsilon _{j})}e^{\beta\tau_{ij}^{+}}+b_{ij}^{+}L_{jg}^{2(1-\varepsilon_{j})}e^{\beta \tau_{ij}^{+}}, \\ \varrho_{2i}(\beta)={}&\beta-2b_{i}+b_{i}^{2} \eta_{i}^{+}+\sum_{j=1}^{n}b_{i} \eta _{i}^{+}b_{ij}^{+}L_{jg}^{2\varepsilon_{j}}+ \sum_{j=1}^{n}b_{ij}^{+}L_{jg}^{2\varepsilon_{j}} \\ & {}+\sum_{i=1}^{n}a_{i}^{2} \delta_{i}^{+}a_{ij}^{+}L_{jf}^{2(1-\xi_{j})}e^{\beta \delta_{ij}^{+}}+a_{ij}^{+}L_{jf}^{2(1-\xi_{j})}e^{\beta\delta _{ij}^{+}}+b_{i} \eta_{i}^{+}e^{\beta\eta_{i}^{+}}. \end{aligned}$$

Obviously, $\varrho_{ji}(\beta)\ (j=1,2;i=1,2,\ldots,n)$ is a continuously differential function. We can easily check that

$$\textstyle\begin{cases} \frac{d\varrho_{1i}(\beta)}{d\beta}=1+\delta_{i}^{+}\sum_{i=1}^{n}a_{i}^{2}\delta_{i}^{+}e^{\beta \delta_{i}^{+}}+\tau_{ij}^{+}\sum_{j=1}^{n}b_{i}\eta _{i}^{+}b_{ij}^{+}L_{jg}^{2(1-\varepsilon_{j})}e^{\beta\tau_{ij}^{+}}+\tau _{ij}^{+}b_{ij}^{+}L_{jg}^{2(1-\varepsilon_{j})}e^{\beta\tau _{ij}^{+}}>0,\\ \lim_{\beta\rightarrow{+\infty}}\varrho_{1i}(\beta)=+\infty,\qquad \varrho _{1i}(0)< 0,\\ \frac{d\varrho_{2i}(\beta)}{d\beta}=1+\delta_{ij}^{+}\sum_{i=1}^{n}a_{i}^{2}\delta_{i}^{+}a_{ij}^{+}L_{jf}^{2(1-\xi_{j})}e^{\beta \delta_{ij}^{+}}+\delta_{ij}^{+}a_{ij}^{+}L_{jf}^{2(1-\xi_{j})}e^{\beta \delta_{ij}^{+}}+\eta_{i}^{+}b_{i}\eta_{i}^{+}e^{\beta\eta_{i}^{+}}>0,\\ \lim_{\beta\rightarrow{+\infty}}\varrho_{2i}(\beta)=+\infty,\qquad \varrho_{2i}(0)< 0. \end{cases} $$

By using the intermediate value theorem, we have that there exist constants $\beta_{i}^{*}>0\ (i=1,2)$ such that

$$\varrho_{j}\bigl(\beta_{i}^{*}\bigr)=0,\quad j=1,2. $$

Let $\beta_{0}=\min\{\beta_{1}^{*},\beta_{2}^{*}\}$, then it follows that $\beta_{0}>0$ and

$$\varrho_{j}(\beta_{0})\leq0,\quad j=1,2. $$

This completes the proof of Lemma 2.2. □

### Lemma 2.3

*Assume that* (H1), (H3), *and* (H4) *are satisfied*. *Then*, *for any solution* $(x_{1}(t),x_{2}(t),\ldots,x_{n}(t), y_{1},y_{2},\ldots, y_{n})^{T}$ *of system* (1.4), *there exists a constant*

$$\begin{aligned} \sigma^{*}={}& \biggl[1-\frac{\sqrt{2}}{\alpha}\bigl(a_{i} \delta_{i}^{+}+b_{i}\eta _{i}^{+} \bigr) \biggr]^{-1} \bigl[a_{i}\delta_{i}^{+} \bigl(a_{ij}^{+}M_{j}^{f}+I_{i}^{+} \bigr) \\ & {}+a_{ij}^{+}M_{j}^{f}+I_{i}^{+}+b_{i} \eta_{i}^{+} \bigl(b_{ij}^{+}M_{j}^{g}+J_{i}^{+} \bigr)+b_{ij}^{+}M_{j}^{g}+J_{i}^{+} \bigr] \end{aligned}$$

*such that*

$$\bigl\vert x_{i}(t) \bigr\vert \leq{\sigma^{*}},\qquad \bigl\vert y_{i}(t) \bigr\vert \leq{\sigma^{*}},\quad i=1,2,\ldots,n, $$

*for all* $t>0$.

### Proof

From (1.4), we have

2.2 $$\begin{aligned} \textstyle\begin{cases} \frac{dx_{i}(t)}{dt}=-a_{i}x_{i}(t)+a_{i}[x_{i}(t)-x_{i}(t-\delta_{i}(t))]+\sum_{j=1}^{n}a_{ij}(t)f_{j}(y_{j}(t-\sigma_{ij}(t)))+I_{i}(t),\\ \frac{dy_{i}(t)}{dt}=-b_{i}y_{i}(t)+b_{i}[y_{i}(t)-y_{i}(t-\eta_{i}(t))]+\sum_{j=1}^{n}b_{ij}(t)g_{j}(x_{j}(t-\tau_{ij}(t)))+J_{i}(t). \end{cases}\displaystyle \end{aligned}$$

Then we have

2.3 $$\begin{aligned} \textstyle\begin{cases} \frac{dx_{i}(t)}{dt}=-a_{i}x_{i}(t)+a_{i}\int_{t-\delta _{i}(t)}^{t}x_{i}^{\prime}(s)\,ds+\sum_{j=1}^{n}a_{ij}(t)f_{j}(y_{j}(t-\sigma _{ij}(t)))+I_{i}(t),\\ \frac{dy_{i}(t)}{dt}=-b_{i}y_{i}(t)+b_{i}\int_{t-\eta_{i}(t)}^{t}y_{i}^{\prime}(s)\,ds+\sum_{j=1}^{n}b_{ij}(t)g_{j}(x_{j}(t-\tau_{ij}(t)))+J_{i}(t). \end{cases}\displaystyle \end{aligned}$$

Thus

2.4 $$\begin{aligned} \textstyle\begin{cases} \frac{dx_{i}(t)}{dt}=-a_{i}x_{i}(t)+a_{i}\int_{t-\delta_{i}(t)}^{t} [-a_{i}x_{i}(s-\delta_{i}(s))\\ \phantom{\frac{dx_{i}(t)}{dt}=}{}+\sum_{j=1}^{n}a_{ij}(s)f_{j}(y_{j}(s-\sigma _{ij}(s)))+I_{i}(s) ]\,ds\\ \phantom{\frac{dx_{i}(t)}{dt}=}{}+\sum_{j=1}^{n}a_{ij}(t)f_{j}(y_{j}(t-\sigma_{ij}(t)))+I_{i}(t),\\ \frac{dy_{i}(t)}{dt}=-b_{i}y_{i}(t)+b_{i}\int_{t-\eta_{i}(t)}^{t} [-b_{i}y_{i}(s-\eta_{i}(s))\\ \phantom{\frac{dy_{i}(t)}{dt}=}{}+\sum_{j=1}^{n}b_{ij}(s)g_{j}(x_{j}(s-\tau _{ij}(s)))+J_{i}(s) ]\,ds\\ \phantom{\frac{dx_{i}(t)}{dt}=}{}+\sum_{j=1}^{n}b_{ij}(t)g_{j}(x_{j}(t-\tau_{ij}(t)))+J_{i}(t). \end{cases}\displaystyle \end{aligned}$$

Let

$$\begin{aligned} &z_{ii}(t)=\left ( \begin{matrix} x_{i}(t) \\ y_{i}(t) \end{matrix} \right ),\qquad A=\left ( \begin{matrix} -a_{i} & 0 \\ 0 & -b_{i} \end{matrix} \right ),\\ & f \bigl(x_{i}(t),y_{i}(t)\bigr)=\left ( \begin{matrix} f_{1}(x_{i}(t),y_{i}(t) \\ f_{2}(x_{i}(t),y_{i}(t) \end{matrix} \right),\qquad I_{ii}(t)= \left( \begin{matrix} I_{i}(t) \\ J_{i}(t) \end{matrix} \right), \end{aligned}$$

where

$$\begin{aligned} f_{1}(x_{i}(t),y_{i}(t)={}&a_{i} \int_{t-\delta_{i}(t)}^{t} \Biggl[-a_{i}x_{i} \bigl(s-\delta _{i}(s)\bigr)+\sum_{j=1}^{n}a_{ij}(s)f_{j} \bigl(y_{j}\bigl(s-\sigma_{ij}(s)\bigr)\bigr)+I_{i}(s) \Biggr]\,ds \\ & {}+\sum_{j=1}^{n}a_{ij}(t)f_{j} \bigl(y_{j}\bigl(t-\sigma_{ij}(t)\bigr)\bigr), \\ f_{2}(x_{i}(t),y_{i}(t)={}& b_{i} \int_{t-\eta_{i}(t)}^{t} \Biggl[-b_{i}y_{i} \bigl(s-\eta _{i}(s)\bigr)+\sum_{j=1}^{n}b_{ij}(s)g_{j} \bigl(x_{j}\bigl(s-\tau_{ij}(s)\bigr)\bigr)+J_{i}(s) \Biggr]\,ds \\ & {}+\sum_{j=1}^{n}b_{ij}(t)g_{j} \bigl(x_{j}\bigl(t-\tau_{ij}(t)\bigr)\bigr), \end{aligned}$$

then system (1.4) can be written in the following equivalent form:

2.5 $$\begin{aligned} z_{ii}^{\prime}(t)\leq{A}z_{ii}(t)+f \bigl(x_{i}(t),y_{i}(t)\bigr)+I_{ii}(t). \end{aligned}$$

Solving inequality (2.5), we have

$$z_{ii}(t)\leq{e^{At}}z_{ii}(0)+ \int_{0}^{t}e^{A(t-s)}\bigl[f \bigl(x_{i}(s),y_{i}(s)\bigr)+I_{ii}(t)\bigr]\,ds. $$

It follows from Lemma 2.1 that

2.6 $$\begin{aligned} \bigl\Vert z_{ii}(t) \bigr\Vert \leq {}&\sqrt{2}e^{-\alpha t} \bigl\Vert z_{ii}(0) \bigr\Vert +\sqrt{2} \int_{0}^{t}e^{\alpha (t-s)}\bigl[ \bigl\Vert f \bigl(x_{i}(s),y_{i}(s)\bigr) \bigr\Vert + \bigl\vert I_{ii}(s) \bigr\vert \bigr]\,ds \\ \leq{}& \sqrt{2} \Vert \varphi \Vert ^{2}+\frac{\sqrt{2}}{\alpha} \bigl(1-e^{-\alpha t} \bigr) \\ &{} \times \bigl[a_{i}\delta_{i}^{+} \bigl( \bigl\vert x_{i}\bigl(t-\delta _{i}(t)\bigr) \bigr\vert +a_{ij}^{+}M_{j}^{f}+I_{i}^{+} \bigr)+a_{ij}^{+}M_{j}^{f}+I_{i}^{+} \\ &{} +b_{i}\eta_{i}^{+} \bigl( \bigl\vert y_{i}\bigl(t-\eta _{i}(t)\bigr) \bigr\vert +b_{ij}^{+}M_{j}^{g}+J_{i}^{+} \bigr)+b_{ij}^{+}M_{j}^{g}+J_{i}^{+} \bigr] \\ \leq {}&\sqrt{2} \Vert \varphi \Vert ^{2}+\frac{\sqrt{2}}{\alpha} \bigl(1-e^{-\alpha t} \bigr) \\ &{} \times \bigl[a_{i}\delta_{i}^{+} \bigl( \bigl\Vert z_{ii}(t) \bigr\Vert +a_{ij}^{+}M_{j}^{f}+I_{i}^{+} \bigr)+a_{ij}^{+}M_{j}^{f}+I_{i}^{+} \\ &{} + b_{i}\eta_{i}^{+} \bigl(\Vert z_{ii}(t) \Vert +b_{ij}^{+}M_{j}^{g}+J_{i}^{+} \bigr)+b_{ij}^{+}M_{j}^{g}+J_{i}^{+} \bigr] \\ \leq{}& \sqrt{2} \Vert \varphi \Vert ^{2}+\frac{\sqrt{2}}{\alpha} \\ &{} \times \bigl[a_{i}\delta_{i}^{+} \bigl( \bigl\Vert z_{ii}(t) \bigr\Vert +a_{ij}^{+}M_{j}^{f}+I_{i}^{+} \bigr)+a_{ij}^{+}M_{j}^{f}+I_{i}^{+} \\ &{} + b_{i}\eta_{i}^{+} \bigl( \bigl\Vert z_{ii}(t) \bigr\Vert +b_{ij}^{+}M_{j}^{g}+J_{i}^{+} \bigr)+b_{ij}^{+}M_{j}^{g}+J_{i}^{+} \bigr]. \end{aligned}$$

Then

$$\begin{aligned} \bigl\Vert z_{ii}(t) \bigr\Vert \leq{}& \biggl[1- \frac{\sqrt{2}}{\alpha}\bigl(a_{i}\delta _{i}^{+}+b_{i} \eta_{i}^{+}\bigr) \biggr]^{-1} \bigl[a_{i}\delta_{i}^{+} \bigl(a_{ij}^{+}M_{j}^{f}+I_{i}^{+} \bigr) \\ & {}+a_{ij}^{+}M_{j}^{f}+I_{i}^{+}+b_{i} \eta_{i}^{+} \bigl(b_{ij}^{+}M_{j}^{g}+J_{i}^{+} \bigr)+b_{ij}^{+}M_{j}^{g}+J_{i}^{+} \bigr]. \end{aligned}$$

Let

$$\begin{aligned} \sigma^{*}={}& \biggl[1-\frac{\sqrt{2}}{\alpha}\bigl(a_{i} \delta_{i}^{+}+b_{i}\eta _{i}^{+} \bigr) \biggr]^{-1} \bigl[a_{i}\delta_{i}^{+} \bigl(a_{ij}^{+}M_{j}^{f}+I_{i}^{+} \bigr) \\ & {}+a_{ij}^{+}M_{j}^{f}+I_{i}^{+}+b_{i} \eta_{i}^{+} \bigl(b_{ij}^{+}M_{j}^{g}+J_{i}^{+} \bigr)+b_{ij}^{+}M_{j}^{g}+J_{i}^{+} \bigr]. \end{aligned}$$

Then it follows that $\vert x_{i}(t) \vert \leq\sigma^{*}, \vert y_{i}(t) \vert \leq\sigma^{*}, i=1,2,\ldots,n$, for all $t>0$. This completes the proof of Lemma 2.3. □

## Main results

In this section, we present our main result that there exists an exponentially stable anti-periodic solution of (1.4).

### Theorem 3.1

*Assume that* (H1)–(H4) *hold true*. *Then any solution* $(x^{*}(t),y^{*}(t))^{T}$ *of system* (1.4) *is globally exponentially stable*.

### Proof

Let $u_{i}(t)=x_{i}(t)-x_{i}^{*}(t), v_{i}(t)=y_{i}(t)-y_{i}^{*}(t), i=1,2,\ldots,n$. It follows from system (2.4) that

3.1 $$\begin{aligned} \textstyle\begin{cases} \frac{du_{i}(t)}{dt}=-a_{i}u_{i}(t)+a_{i}\int_{t-\delta_{i}(t)}^{t} [-a_{i}u_{i}(s-\delta_{i}(s))\\ \phantom{\frac{du_{i}(t)}{dt}=}{}+\sum_{j=1}^{n}a_{ij}(s)(f_{j}(y_{j}(s-\sigma_{ij}(s)))-f_{j}(y_{j}^{*}(s-\sigma _{ij}(s)))) ]\,ds\\ \phantom{\frac{du_{i}(t)}{dt}=}{}+\sum_{j=1}^{n}a_{ij}(t)(f_{j}(y_{j}(t-\sigma_{ij}(t)))-f_{j}(y_{j}^{*}(t-\sigma _{ij}(t)))),\\ \frac{dv_{i}(t)}{dt}=-b_{i}v_{i}(t)+b_{i}\int_{t-\eta_{i}(t)}^{t} [-b_{i}v_{i}(s-\eta_{i}(s))\\ \phantom{\frac{dv_{i}(t)}{dt}=}{}+\sum_{j=1}^{n}b_{ij}(s)(g_{j}(x_{j}(s-\tau_{ij}(s)))-g_{j}(x_{j}^{*}(s-\tau _{ij}(s)))) ]\,ds\\ \phantom{\frac{dv_{i}(t)}{dt}=}{}+\sum_{j=1}^{n}b_{ij}(t)(g_{j}(x_{j}(t-\tau_{ij}(t)))-g_{j}(x_{j}(t-\tau_{ij}(t)))), \end{cases}\displaystyle \end{aligned}$$

which leads to

3.2 $$\begin{aligned} \textstyle\begin{cases} \frac{1}{2}\frac{du_{i}^{2}(t)}{dt}=-a_{i}u_{i}^{2}(t)+a_{i}u_{i}(t)\int_{t-\delta _{i}(t)}^{t} [-a_{i}u_{i}(s-\delta_{i}(s))\\ \phantom{\frac{1}{2}\frac{du_{i}^{2}(t)}{dt}=}{}+\sum_{j=1}^{n}a_{ij}(s)(f_{j}(y_{j}(s-\sigma_{ij}(s)))-f_{j}(y_{j}^{*}(s-\sigma _{ij}(s)))) ]\,ds\\ \phantom{\frac{1}{2}\frac{du_{i}^{2}(t)}{dt}=}{}+u_{i}(t)\sum_{j=1}^{n}a_{ij}(t)(f_{j}(y_{j}(t-\sigma _{ij}(t)))-f_{j}(y_{j}^{*}(t-\sigma_{ij}(t)))),\\ \frac{1}{2}\frac{dv_{i}^{2}(t)}{dt}=-b_{i}v_{i}^{2}(t)+b_{i}v_{i}(t)\int_{t-\eta _{i}(t)}^{t} [-b_{i}v_{i}(s-\eta_{i}(s))\\ \phantom{\frac{1}{2}\frac{dv_{i}^{2}(t)}{dt}=}{}+\sum_{j=1}^{n}b_{ij}(s)(g_{j}(x_{j}(s-\tau_{ij}(s)))-g_{j}(x_{j}^{*}(s-\tau _{ij}(s)))) ]\,ds\\ \phantom{\frac{1}{2}\frac{dv_{i}^{2}(t)}{dt}=}{}+v_{i}(t)\sum_{j=1}^{n}b_{ij}(t)(g_{j}(x_{j}(t-\tau_{ij}(t)))-g_{j}(x_{j}(t-\tau_{ij}(t)))). \end{cases}\displaystyle \end{aligned}$$

In view of condition (H1), we get

3.3 $$\begin{aligned} \textstyle\begin{cases} \frac{du_{i}^{2}(t)}{dt}\leq-2a_{i}u_{i}^{2}(t)+a_{i}u_{i}(t)\int_{t-\delta _{i}(t)}^{t} [-a_{i}u_{i}(s-\delta_{i}(s))\\ \phantom{\frac{du_{i}^{2}(t)}{dt}\leq}{}+\sum_{j=1}^{n}a_{ij}(s)(f_{j}(y_{j}(s-\sigma_{ij}(s)))-f_{j}(y_{j}^{*}(s-\sigma _{ij}(s)))) ]\,ds\\ \phantom{\frac{du_{i}^{2}(t)}{dt}\leq}{}+u_{i}(t)\sum_{j=1}^{n}a_{ij}(t)(f_{j}(y_{j}(t-\sigma _{ij}(t)))-f_{j}(y_{j}^{*}(t-\sigma_{ij}(t)))),\\ \frac{dv_{i}^{2}(t)}{dt}\leq-2b_{i}v_{i}^{2}(t)+b_{i}v_{i}(t)\int_{t-\eta_{i}(t)}^{t} [-b_{i}v_{i}(s-\eta_{i}(s))\\ \phantom{\frac{dv_{i}^{2}(t)}{dt}\leq}{}+\sum_{j=1}^{n}b_{ij}(s)(g_{j}(x_{j}(s-\tau_{ij}(s)))-g_{j}(x_{j}^{*}(s-\tau _{ij}(s)))) ]\,ds\\ \phantom{\frac{dv_{i}^{2}(t)}{dt}\leq}{}+v_{i}(t)\sum_{j=1}^{n}b_{ij}(t)(g_{j}(x_{j}(t-\tau_{ij}(t)))-g_{j}(x_{j}(t-\tau_{ij}(t)))). \end{cases}\displaystyle \end{aligned}$$

Then

3.4 $$\begin{aligned} \textstyle\begin{cases} \frac{du_{i}^{2}(t)}{dt}\leq-2a_{i}u_{i}^{2}(t)+a_{i}\delta_{i}^{+} [a_{i}(u_{i}^{2}+u_{i}^{2}(t-\delta_{i}(t)))\\ \phantom{\frac{du_{i}^{2}(t)}{dt}\leq}{}+\sum_{j=1}^{n}a_{ij}^{+}(L_{jf}^{2\xi_{j}}u_{i}^{2}(t)+L_{jf}^{2(1-\xi _{j})}v_{j}^{2}(t-\delta_{ij}(t))) ]\\ \phantom{\frac{du_{i}^{2}(t)}{dt}\leq}{}+\sum_{j=1}^{n}a_{ij}^{+}(L_{jf}^{2\xi_{j}}u_{i}^{2}(t)+L_{jf}^{2(1-\xi _{j})}v_{j}^{2}(t-\delta_{ij}(t))),\\ \frac{dv_{i}^{2}(t)}{dt}\leq-2b_{i}v_{i}^{2}(t)+b_{i}\eta_{i}^{+} [b_{i}(v_{i}^{2}+v_{i}^{2}(t-\eta_{i}(t)))\\ \phantom{\frac{dv_{i}^{2}(t)}{dt}\leq}{}+\sum_{j=1}^{n}b_{ij}^{+}(L_{jg}^{2\varepsilon _{j}}v_{i}^{2}(t)+L_{jg}^{2(1-\varepsilon_{j})}u_{j}^{2}(t-\tau_{ij}(t))) ]\\ \phantom{\frac{dv_{i}^{2}(t)}{dt}\leq}{}+\sum_{j=1}^{n}b_{ij}^{+}(L_{jg}^{\varepsilon _{j}}v_{i}^{2}(t)+L_{jg}^{2(1-\varepsilon_{j})}u_{j}^{2}(t-\tau_{ij}(t))), \end{cases}\displaystyle \end{aligned}$$

where $0\leq\xi_{j},\varepsilon_{j}\leq1,j=1,2,\ldots,n$.

Now we consider the following Lyapunov function:

3.5 $$\begin{aligned} V(t)={}&e^{\beta t}\sum_{i=1}^{n}u_{i}^{2}(t)+e^{\beta t} \sum_{i=1}^{n}v_{i}^{2}(t) \\ &{}+\sum_{i=1}^{n}a_{i}^{2} \delta_{i}^{+} \int_{t-\delta_{i}(t)}^{t}e^{\beta(s+\delta _{i}(t))}u_{i}^{2}(s)\,ds \\ & {}+\sum_{i=1}^{n}\sum _{j=1}^{n}a_{i}\delta_{i}^{+}a_{ij}^{+}L_{jf}^{2(1-\xi _{j})} \int_{t-\delta_{ij}(t)}^{t}e^{\beta(s+\delta_{ij}(t))}v_{j}^{2}(s)\,ds \\ & {}+\sum_{i=1}^{n}a_{ij}^{+}L_{jf}^{2(1-\xi_{j})} \int_{t-\delta _{ij}(t)}^{t}e^{\beta(s+\delta_{ij}(t))}v_{j}^{2}(s)\,ds \\ & {}+\sum_{i=1}^{n}b_{i}^{2} \eta_{i}^{+} \int_{t-\eta_{i}(t)}^{t}e^{\beta(s+\eta _{i}(t))}v_{i}^{2}(s)\,ds \\ & {}+\sum_{i=1}^{n}\sum _{j=1}^{n}b_{i}\eta _{i}^{+}b_{ij}^{+}L_{jg}^{2(1-\varepsilon_{j})} \int_{t-\tau _{ij}(t)}^{t}e^{\beta(s+\tau_{ij}(t))}u_{j}^{2}(s)\,ds \\ & {}+\sum_{i=1}^{n}b_{ij}^{+}L_{jg}^{2(1-\varepsilon_{j})} \int_{t-\tau _{ij}(t)}^{t}e^{\beta(s+\tau_{ij}(t))}u_{j}^{2}(s)\,ds, \end{aligned}$$

where *β* is given by Lemma 2.2. Differentiating $V(t)$ along solutions to system (1.4), together with (3.3), we have

3.6 $$\begin{aligned} \frac{dV(t)}{dt}\leq{}&\beta{e^{\beta t}} \Biggl[\sum _{i=1}^{n}u_{i}^{2}(t)+\sum _{i=1}^{n}v_{i}^{2}(t) \Biggr] \\ & {}+e^{\beta t}\sum_{j=1}^{n} \Biggl\{ -2a_{i}u_{i}^{2}(t)+a_{i} \delta_{i}^{+} \Biggl[a_{i}\bigl(u_{i}^{2}+u_{i}^{2} \bigl(t-\delta_{i}(t)\bigr)\bigr) \\ & {}+\sum_{j=1}^{n}a_{ij}^{+} \bigl(L_{jf}^{2\xi_{j}}u_{i}^{2}(t)+L_{jf}^{2(1-\xi _{j})}v_{j}^{2} \bigl(t-\delta_{ij}(t)\bigr)\bigr) \Biggr] \\ & {}+\sum_{j=1}^{n}a_{ij}^{+} \bigl(L_{jf}^{2\xi_{j}}u_{i}^{2}(t)+L_{jf}^{2(1-\xi _{j})}v_{j}^{2} \bigl(t-\delta_{ij}(t)\bigr)\bigr) \Biggr\} \\ & {}+e^{\beta t}\sum_{j=1}^{n} \Biggl\{ -2b_{i}v_{i}^{2}(t)+b_{i} \eta_{i}^{+} \Biggl[b_{i}\bigl(v_{i}^{2}+v_{i}^{2} \bigl(t-\eta_{i}(t)\bigr)\bigr) \\ & {}+\sum_{j=1}^{n}b_{ij}^{+} \bigl(L_{jg}^{2\varepsilon _{j}}v_{i}^{2}(t)+L_{jg}^{2(1-\varepsilon_{j})}u_{j}^{2} \bigl(t-\tau_{ij}(t)\bigr)\bigr) \Biggr] \\ &{} +\sum_{j=1}^{n}b_{ij}^{+} \bigl(L_{jg}^{\varepsilon _{j}}v_{i}^{2}(t)+L_{jg}^{2(1-\varepsilon_{j})}u_{j}^{2} \bigl(t-\tau_{ij}(t)\bigr)\bigr) \Biggr\} \\ & {}+\sum_{i=1}^{n}a_{i}^{2} \delta_{i}^{+}\bigl[e^{\beta(t+\delta_{i}(t))} u_{i}^{2}(t)-e^{\beta t}u_{i}^{2} \bigl(t-\delta_{i}(t)\bigr)\bigr] \\ & {}+\sum_{i=1}^{n}\sum _{j=1}^{n}a_{i}\delta_{i}^{+}a_{ij}^{+}L_{jf}^{2(1-\xi _{j})} \bigl[e^{\beta(t+\delta_{ij}(t))}v_{j}^{2}(t)-e^{\beta t}v_{j}^{2} \bigl(t-\delta _{ij}(t)\bigr)\bigr] \\ & {}+\sum_{i=1}^{n}a_{ij}^{+}L_{jf}^{2(1-\xi_{j})} \bigl[e^{\beta(t+\delta _{ij}(t))}v_{j}^{2}(t)-e^{\beta(t)}v_{j}^{2} \bigl(t-\delta_{ij}(t)\bigr)\bigr] \\ & {}+\sum_{i=1}^{n}b_{i}^{2} \eta_{i}^{+}\bigl[e^{\beta(t+\eta_{i}(t))}v_{i}^{2}(t)-e^{\beta t}v_{i}^{2} \bigl(t-\eta_{i}(t)\bigr)\bigr] \\ & {}+\sum_{i=1}^{n}\sum _{j=1}^{n}b_{i}\eta _{i}^{+}b_{ij}^{+}L_{jg}^{2(1-\varepsilon_{j})} \bigl[e^{\beta(t+\tau _{ij}(t))}u_{j}^{2}(t)-e^{\beta t}u_{j}^{2} \bigl(t-\tau_{ij}(t)\bigr)\bigr] \\ & {}+\sum_{i=1}^{n}b_{ij}^{+}L_{jg}^{2(1-\varepsilon_{j})} \bigl[e^{\beta(t+\tau _{ij}(t))}u_{j}^{2}(t)-e^{\beta t}u_{j}^{2} \bigl(t-\tau_{ij}(t)\bigr)\bigr] \\ ={}&e^{\beta t} \Biggl[\beta-2a_{i}+a_{i}^{2} \delta_{i}^{+}+\sum_{j=1}^{n}a_{i} \delta _{i}^{+}a_{ij}^{+}L_{jf}^{2\xi_{j}}+ \sum_{j=1}^{n}a_{ij}^{+}L_{jf}^{2\xi _{j}}+ \sum_{i=1}^{n}a_{i}^{2} \delta_{i}^{+}e^{\beta\delta_{i}(t)} \\ & {}+\sum_{j=1}^{n}b_{i} \eta_{i}^{+}b_{ij}^{+}L_{jg}^{2(1-\varepsilon _{j})}e^{\beta\tau_{ij}(t)}+b_{ij}^{+}L_{jg}^{2(1-\varepsilon_{j})}e^{\beta \tau_{ij}(t)} \Biggr]x_{i}^{2}(t) \\ & {}+e^{\beta t} \Biggl[\beta-2b_{i}+b_{i}^{2} \eta_{i}^{+}+\sum_{j=1}^{n}b_{i} \eta _{i}^{+}b_{ij}^{+}L_{jg}^{2\varepsilon_{j}}+ \sum_{j=1}^{n}b_{ij}^{+}L_{jg}^{2\varepsilon_{j}} \\ & {}+\sum_{i=1}^{n}a_{i}^{2} \delta_{i}^{+}a_{ij}^{+}L_{jf}^{2(1-\xi_{j})}e^{\beta \delta_{ij}(t)}+a_{ij}^{+}L_{jf}^{2(1-\xi_{j})}e^{\beta\delta _{ij}(t)}+b_{i} \eta_{i}^{+}e^{\beta\eta_{i}(t)} \Biggr]y_{i}^{2}(t) \\ \leq{}&e^{\beta t} \Biggl[\beta-2a_{i}+a_{i}^{2} \delta_{i}^{+}+\sum_{j=1}^{n}a_{i} \delta _{i}^{+}a_{ij}^{+}L_{jf}^{2\xi_{j}}+ \sum_{j=1}^{n}a_{ij}^{+}L_{jf}^{2\xi _{j}}+ \sum_{i=1}^{n}a_{i}^{2} \delta_{i}^{+}e^{\beta\delta_{i}^{+}} \\ & {}+\sum_{j=1}^{n}b_{i} \eta_{i}^{+}b_{ij}^{+}L_{jg}^{2(1-\varepsilon _{j})}e^{\beta\tau_{ij}^{+}}+b_{ij}^{+}L_{jg}^{2(1-\varepsilon_{j})}e^{\beta \tau_{ij}^{+}} \Biggr]x_{i}^{2}(t) \\ & {}+e^{\beta t} \Biggl[\beta-2b_{i}+b_{i}^{2} \eta_{i}^{+}+\sum_{j=1}^{n}b_{i} \eta _{i}^{+}b_{ij}^{+}L_{jg}^{2\varepsilon_{j}}+ \sum_{j=1}^{n}b_{ij}^{+}L_{jg}^{2\varepsilon_{j}} \\ & {}+\sum_{i=1}^{n}a_{i}^{2} \delta_{i}^{+}a_{ij}^{+}L_{jf}^{2(1-\xi_{j})}e^{\beta \delta_{ij}^{+}}+a_{ij}^{+}L_{jf}^{2(1-\xi_{j})}e^{\beta\delta _{ij}^{+}}+b_{i} \eta_{i}^{+}e^{\beta\eta_{i}^{+}} \Biggr]y_{i}^{2}(t). \end{aligned}$$

It follows from Lemma 2.2 that $\frac{dV(t)}{dt}\leq0$, which implies that $V(t)\leq{V(0)}$ for all $t>0$. Thus

3.7 $$\begin{aligned} & e^{\beta t} \Biggl[\sum_{i=1}^{n}u_{i}^{2}(t)+ \sum_{i=1}^{n}v_{i}^{2}(t) \Biggr] \\ &\quad \leq\sum_{i=1}^{n}u_{i}^{2}(0)+ \sum_{i=1}^{n}v_{i}^{2}(0) \\ & \qquad{}+\sum_{i=1}^{n}a_{i}^{2} \delta_{i}^{+} \int_{-\delta_{i}(0)}^{0}e^{\beta(s+\delta _{i}(0))}u_{i}^{2}(s)\,ds \\ & \qquad{}+\sum_{i=1}^{n}\sum _{j=1}^{n}a_{i}\delta_{i}^{+}a_{ij}^{+}L_{jf}^{2(1-\xi _{j})} \int_{-\delta_{ij}(0)}^{0}e^{\beta(s+\delta_{ij}(0))}v_{j}^{2}(s)\,ds \\ & \qquad{}+\sum_{i=1}^{n}a_{ij}^{+}L_{jf}^{2(1-\xi_{j})} \int_{-\delta _{ij}(0)}^{0}e^{\beta(s+\delta_{ij}(0))}v_{j}^{2}(s)\,ds \\ & \qquad{}+\sum_{i=1}^{n}b_{i}^{2} \eta_{i}^{+} \int_{-\eta_{i}(0)}^{0}e^{\beta(s+\eta _{i}(0))}v_{i}^{2}(s)\,ds \\ & \qquad{}+\sum_{i=1}^{n}\sum _{j=1}^{n}b_{i}\eta _{i}^{+}b_{ij}^{+}L_{jg}^{2(1-\varepsilon_{j})} \int_{-\tau _{ij}(0)}^{0}e^{\beta(s+\tau_{ij}(0))}u_{j}^{2}(s)\,ds \\ & \qquad{}+\sum_{i=1}^{n}b_{ij}^{+}L_{jg}^{2(1-\varepsilon_{j})} \int_{-\tau _{ij}(0)}^{t}e^{\beta(s+\tau_{ij}(0))}u_{j}^{2}(s)\,ds \\ &\quad \leq \bigl\Vert \varphi-\varphi^{*} \bigr\Vert ^{2}+ \bigl\Vert \psi-\psi^{*} \bigr\Vert ^{2}+\sum_{i=1}^{n}a_{i}^{2} \delta _{i}^{+}\frac{1}{\beta}e^{\beta\delta_{i}^{+}} \bigl\Vert \varphi-\varphi ^{*} \bigr\Vert ^{2} \\ & \qquad{}+\sum_{i=1}^{n}\sum _{j=1}^{n}a_{i}\delta_{i}^{+}a_{ij}^{+}L_{jf}^{2(1-\xi _{j})} \frac{1}{\beta}e^{\beta\delta_{ij}^{+}} \bigl\Vert \psi-\psi^{*} \bigr\Vert ^{2} \\ & \qquad{}+\sum_{i=1}^{n}a_{ij}^{+}L_{jf}^{2(1-\xi_{j})} \frac{1}{\beta}e^{\beta \delta_{ij}^{+}} \bigl\Vert \psi-\psi^{*} \bigr\Vert ^{2} \\ & \qquad{}+\sum_{i=1}^{n}b_{i}^{2} \eta_{i}^{+}\frac{1}{\beta}e^{\beta\eta _{i}^{+}} \bigl\Vert \psi-\psi^{*} \bigr\Vert ^{2} \\ & \qquad{}+\sum_{i=1}^{n}\sum _{j=1}^{n}b_{i}\eta _{i}^{+}b_{ij}^{+}L_{jg}^{2(1-\varepsilon_{j})} \frac{1}{\beta}e^{\beta\tau _{ij}^{+}} \bigl\Vert \varphi-\varphi^{*} \bigr\Vert ^{2} \\ & \qquad{}+\sum_{i=1}^{n}b_{ij}^{+}L_{jg}^{2(1-\varepsilon_{j})} \frac{1}{\beta }e^{\beta\tau_{ij}^{+}} \bigl\Vert \varphi-\varphi^{*} \bigr\Vert ^{2} \\ &\quad= \Biggl[1+\sum_{i=1}^{n}a_{i}^{2} \delta_{i}^{+}\frac{1}{\beta}e^{\beta\delta _{i}^{+}}+\sum _{i=1}^{n}\sum_{j=1}^{n}b_{i} \eta _{i}^{+}b_{ij}^{+}L_{jg}^{2(1-\varepsilon_{j})} \frac{1}{\beta}e^{\beta\tau _{ij}^{+}} \\ & \qquad{} +\sum_{i=1}^{n}b_{ij}^{+}L_{jg}^{2(1-\varepsilon_{j})} \frac {1}{\beta}e^{\beta\tau_{ij}^{+}} \Biggr] \bigl\Vert \varphi-\varphi^{*} \bigr\Vert ^{2} \\ & \qquad{}+ \Biggl[1+\sum_{i=1}^{n}\sum _{j=1}^{n}a_{i}\delta _{i}^{+}a_{ij}^{+}L_{jf}^{2(1-\xi_{j})} \frac{1}{\beta}e^{\beta\delta _{ij}^{+}} \\ & \qquad{}+\sum_{i=1}^{n}a_{ij}^{+}L_{jf}^{2(1-\xi_{j})} \frac{1}{\beta}e^{\beta \delta_{ij}^{+}}+\sum_{i=1}^{n}b_{i}^{2} \eta_{i}^{+}\frac{1}{\beta }e^{\beta\eta_{i}^{+}} \Biggr] \bigl\Vert \psi-\psi^{*} \bigr\Vert ^{2}. \end{aligned}$$

Let

$$\begin{aligned} &\theta_{1}=1+\sum_{i=1}^{n}a_{i}^{2} \delta_{i}^{+}\frac{1}{\beta}e^{\beta\delta _{i}^{+}}+\sum _{i=1}^{n}\sum_{j=1}^{n}b_{i} \eta _{i}^{+}b_{ij}^{+}L_{jg}^{2(1-\varepsilon_{j})} \frac{1}{\beta}e^{\beta\tau_{ij}^{+}} +\sum_{i=1}^{n}b_{ij}^{+}L_{jg}^{2(1-\varepsilon_{j})} \frac{1}{\beta }e^{\beta\tau_{ij}^{+}}, \\ &\theta_{2}=1+\sum_{i=1}^{n}\sum _{j=1}^{n}a_{i}\delta _{i}^{+}a_{ij}^{+}L_{jf}^{2(1-\xi_{j})} \frac{1}{\beta}e^{\beta\delta _{ij}^{+}}+\sum_{i=1}^{n}a_{ij}^{+}L_{jf}^{2(1-\xi_{j})} \frac{1}{\beta }e^{\beta\delta_{ij}^{+}} \sum_{i=1}^{n}b_{i}^{2} \eta_{i}^{+}\frac{1}{\beta}e^{\beta\eta_{i}^{+}} \end{aligned}$$

and choose

$$M=\max\{\theta_{1},\theta_{2}\}>1. $$

Then Eq. (3.7) can be rewritten as

$$\sum_{i=1}^{n}u_{i}^{2}(t)+ \sum_{i=1}^{n}v_{i}^{2}(t) \leq{M}e^{-\beta t} \bigl[ \bigl\Vert \varphi-\varphi^{*} \bigr\Vert ^{2}+ \bigl\Vert \psi-\psi^{*} \bigr\Vert ^{2} \bigr] $$

for all $t>0$. Then

$$\sum_{i=1}^{n} \bigl\vert x_{i}(t)-x_{i}^{*}(t) \bigr\vert ^{2}+\sum _{i=1}^{n} \bigl\vert y_{i}(t)-y_{i}^{*}(t) \bigr\vert ^{2}\leq {M}e^{-\beta t} \bigl[ \bigl\Vert \varphi- \varphi^{*} \bigr\Vert ^{2}+ \bigl\Vert \psi-\psi^{*} \bigr\Vert ^{2} \bigr] $$

for all $t>0$. Thus the solution $(x(t),y(t))^{T}$ of system (1.4) is globally exponentially stable. □

### Theorem 3.2

*Assume that* (H1)–(H4) *are satisfied*. *Then system* (1.4) *has exactly one* *T*-*anti*-*periodic solution which is globally stable*.

### Proof

It follows from system (1.4) and (H2) that for each $k\in{N}$, we have

3.8 $$\begin{aligned} & \frac{d}{dt} \bigl[(-1)^{k+1}x_{i}\bigl(t+(k+1)T \bigr) \bigr] \\ & \quad=(-1)^{k+1} [-a_{i}x_{i}\bigl(t+(k+1)T- \delta_{i}\bigl(t+(k+1)T\bigr)\bigr) \\ & \qquad{}+\sum_{j=1}^{n}a_{ij} \bigl(t+(k+1)T\bigr)f_{j}\bigl(y_{j}\bigl(t+(k+1)T-\sigma _{ij}\bigl(t+(k+1)T\bigr)\bigr)\bigr) \\ & \qquad{}+I_{i}\bigl(t+(k+1)T\bigr) \\ & \quad=-a_{i}(-1)^{k+1}x_{i}\bigl(t+(k+1)T- \delta_{i}(t)\bigr) \\ & \qquad{}+\sum_{j=1}^{n}a_{ij}(t)f_{j} \bigl((-1)^{k+1}y_{j}\bigl(t+(k+1)T-\sigma _{ij}(t) \bigr)\bigr)+I_{i}(t), \end{aligned}$$

3.9 $$\begin{aligned} & \frac{d}{dt} \bigl[(-1)^{k+1}y_{i} \bigl(t+(k+1)T\bigr) \bigr] \\ & \quad=(-1)^{k+1} \Biggl[-b_{i}y_{i}\bigl(t+(k+1)T- \eta_{i}\bigl(t+(k+1)T\bigr)\bigr) \\ & \qquad{}+\sum_{j=1}^{n}b_{ij} \bigl(t+(k+1)T\bigr)g_{j}\bigl(x_{j}\bigl(t+(k+1)T-\tau _{ij}\bigl(t+(k+1)T\bigr)\bigr)\bigr) \\ & \qquad{}+J_{i}\bigl(t+(k+1)T\bigr) \Biggr] \\ & \quad=-b_{i}(-1)^{k+1}y_{i}\bigl(t+(k+1)T- \eta_{i}(t)\bigr) \\ & \qquad{}+\sum_{j=1}^{n}b_{ij}(t)g_{j} \bigl((-1)^{k+1}x_{j}\bigl(t+(k+1)T-\tau _{ij}(t) \bigr)\bigr)+J_{i}(t). \end{aligned}$$

Let

$$\begin{aligned} &\bar{x}(t)=\bigl((-1)^{k+1}x_{1}\bigl(t+(k+1)T \bigr),(-1)^{k+1}x_{2}\bigl(t+(k+1)T\bigr),\ldots ,(-1)^{k+1}x_{n}\bigl(t+(k+1)T\bigr)\bigr)^{T}, \\ &\bar{y}(t)=\bigl((-1)^{k+1}y_{1}\bigl(t+(k+1)T \bigr),(-1)^{k+1}y_{2}\bigl(t+(k+1)T\bigr),\ldots ,(-1)^{k+1}y_{n}\bigl(t+(k+1)T\bigr)\bigr)^{T}. \end{aligned}$$

Obviously, for any $k\in{N},(\bar{x}(t), \bar{y}(t))$ is also a solution of system (1.4). If the initial function $\varphi_{i}(s), \psi_{i}(s)\ (i=1,2,\ldots,n)$ is bounded, it follows from Theorem 3.1 that there exists a constant $\gamma>1$ such that

3.10 $$\begin{aligned} & \bigl\vert (-1)^{k+1}x_{i}\bigl(t+(k+1)T \bigr)-(-1)^{k}x_{i}(t+kT) \bigr\vert \\ & \quad \leq{M}e^{-\beta(t+kT)}\sup_{-\tau\leq{s}\leq0}\sum _{i=1}^{4} \bigl\vert x_{i}(t+T)+x_{i}(s) \bigr\vert ^{2} \\ & \quad \leq\gamma e^{-\beta(t+kT)}, \end{aligned}$$

where $t+kT>0,i=1,2,\ldots,n$. Since for any $k\in{N}$ we have

3.11 $$\begin{aligned} (-1)^{k+1} x_{i} \bigl(t + (k+1)T\bigr) = x_{i} (t ) +\sum_{j=0}^{k}\bigl[(-1)^{j+1} x _{i}\bigl(t + (j+1)T\bigr)-(-1)^{j} x_{i} (t + jT)\bigr], \end{aligned}$$

then

3.12 $$\begin{aligned} (-1)^{k+1} x_{i} \bigl(t + (k+1)T\bigr) \leq \bigl\vert x_{i} (t ) \bigr\vert +\sum_{j=0}^{k} \bigl\vert (-1)^{j+1} x _{i}\bigl(t + (j+1)T \bigr)-(-1)^{j} x_{i} (t + jT) \bigr\vert . \end{aligned}$$

By Lemma 2.3, we know that the solutions of system (1.4) are bounded. In view of (3.10) and (3.12), we can easily know that $\{(-1)^{k+1} x_{i}(t + (k+1)T)\}$ uniformly converges to a continuous function $x^{*}(t)=(x^{*}_{1}(t),x^{*}_{2}(t), \ldots, x^{*}_{n}(t))^{T}$ on any compact set of ${R^{n}}$. In a similar way, we can easily prove that $\{(-1)^{k+1} y_{i}(t + (k+1)T)\}$ uniformly converges to a continuous function $y^{*}(t)=(y^{*}_{1}(t),y^{*}_{2}(t), \ldots, y^{*}_{n}(t))^{T}$ on any compact set of ${R^{n}}$.

Now we show that $x^{*}(t)$ is a *T*-anti-periodic solution of (1.4). Firstly, $x^{*}(t)$ is *T*-anti-periodic since

3.13 $$\begin{aligned} x^{*}(t+T) &=\lim_{k\to\infty}(-1)^{k }x(t +T+ kT) \\ &=-\lim_{(k+1)\to\infty}(-1)^{k+1 } x\bigl(t +(k +1)T \bigr)=-x^{*}(t ). \end{aligned}$$

Then we can conclude that $x_{i}^{*}(t)$ is *T*-anti-periodic on *R*. Similarly, $y_{i}^{*}(t)$ is also *T*-anti-periodic on *R*. Thus we can conclude that $(x^{*}(t),y^{*}(t))^{T}$ is the solution of system (1.4).

In fact, together with the continuity of the right-hand side of system (1.4), let $k\to\infty$, we can easily get

3.14 $$\begin{aligned} \textstyle\begin{cases} \frac{dx_{i}^{*}(t)}{dt}=-a_{i}x_{i}^{*}(t-\delta_{i}(t))+\sum_{j=1}^{n}a_{ij}(t)f_{j}(y_{j}^{*}(t-\sigma_{ij}(t)))+I_{i}(t),\\ \frac{dy_{i}^{*}(t)}{dt}=-b_{i}y_{i}^{*}(t-\eta_{i}(t))+\sum_{j=1}^{n}b_{ij}(t)g_{j}(x_{j}^{*}(t-\tau_{ij}(t)))+J_{i}(t). \end{cases}\displaystyle \end{aligned}$$

Therefore, $(x^{*}(t),y^{*}(t))^{T}$ is a solution of (1.4). Finally, by applying Theorem 3.1, it is easy to check that $(x^{*}(t),y^{*}(t))^{T}$ is globally exponentially stable. This completes the proof of Theorem 3.2. □

## An example

In this section, we give an example to illustrate our main results derived in the previous sections. Consider the following BAM neural network with time-varying delays in the leakage terms:

4.1 $$\begin{aligned} \textstyle\begin{cases} \frac{dx_{1}(t)}{dt}=-a_{1}x_{1}(t-\delta_{1}(t))+\sum_{j=1}^{2}a_{1j}(t)f_{j}(y_{j}(t-\sigma_{1j}(t)))+I_{1}(t),\\ \frac{dx_{2}(t)}{dt}=-a_{2}x_{2}(t-\delta_{2}(t))+\sum_{j=1}^{2}a_{2j}(t)f_{j}(y_{j}(t-\sigma_{2j}(t)))+I_{2}(t),\\ \frac{dy_{1}(t)}{dt}=-b_{1}y_{1}(t-\eta_{1}(t))+\sum_{j=1}^{2}b_{1j}(t)g_{j}(x_{j}(t-\tau_{1j}(t)))+J_{1}(t),\\ \frac{dy_{2}(t)}{dt}=-b_{2}y_{2}(t-\eta_{2}(t))+\sum_{j=1}^{2}b_{2j}(t)g_{j}(x_{j}(t-\tau_{2j}(t)))+J_{2}(t), \end{cases}\displaystyle \end{aligned}$$

where

$$\begin{aligned} & \left [ \begin{matrix} \delta_{1}(t) & \delta_{2}(t) \\ \eta_{1}(t)& \eta_{2}(t) \end{matrix} \right ]=\left [ \begin{matrix} 0.05 \vert \sin t \vert & 0.05 \vert \sin t \vert \\ 0.04 \vert \cos t \vert & 0.04 \vert \cos t \vert \end{matrix} \right ], \\ & \left [ \begin{matrix} a_{11}(t) & a_{12}(t) \\ a_{21}(t)& a_{22}(t) \end{matrix} \right ]=\left [ \begin{matrix} 0.3 \vert \cos t \vert & 0.3 \vert \cos t \vert \\ 0.5 \vert \sin t \vert & 0.5 \vert \sin t \vert \end{matrix} \right ], \\ &\left [ \begin{matrix} b_{11}(t) & b_{12}(t) \\ b_{21}(t)& b_{22}(t) \end{matrix} \right ]=\left [ \begin{matrix} 0.03 \vert \cos t \vert & 0.03 \vert \cos t \vert \\ 0.05 \vert \sin t \vert & 0.05 \vert \sin t \vert \end{matrix} \right ], \\ & \left [ \begin{matrix} I_{1}(t) & I_{2}(t) \\ J_{1}(t)& J_{2}(t) \end{matrix} \right ]=\left [ \begin{matrix} 0.5\cos t& 0.5\cos t \\ 0.5\sin t & 0.5\sin t \end{matrix} \right ],\qquad \left [ \begin{matrix} a_{1} & a_{2} \\ b_{1}& b_{2} \end{matrix} \right ]=\left [ \begin{matrix} 2& 2 \\ 2 & 2 \end{matrix} \right ]. \end{aligned}$$

Set $f_{j}(u)=g_{j}(u)=\frac{1}{2}( \vert u+1 \vert - \vert u-1 \vert ),j=1,2$. Then $L_{jf}=g_{jg}=M_{j}^{f}=M_{j}^{g}=1, \delta_{1}^{+}=\delta_{2}^{+}=0.05, a_{1j}^{+}=0.3, a_{2j}^{+}=0.5, \eta_{1}^{+}=\eta_{2}^{+}=0.04, b_{1j}^{+}=0.03, b_{2j}^{+}=0.05, j=1,2$. It is easy to verify that

$$\frac{\sqrt{2}}{\alpha}\bigl(a_{i}\delta_{i}^{+}+b_{i} \eta_{i}^{+}\bigr)\approx0.12726< 1 $$

and

$$\begin{aligned} & {-}2a_{1}+a_{1}^{2}\delta_{1}^{+}+ \sum_{j=1}^{2}a_{1}\delta _{1}^{+}a_{1j}^{+}L_{jf}^{2\xi_{j}}+ \sum_{j=1}^{2}a_{1j}^{+}L_{jf}^{2\xi_{j}} \\ & \quad{}+\sum_{i=1}^{2}a_{i}^{2} \delta_{i}^{+}+\sum_{j=1}^{2}b_{1} \eta _{1}^{+}b_{1j}^{+}L_{jg}^{2(1-\varepsilon _{j})}+b_{1j}^{+}L_{jg}^{2(1-\varepsilon_{j})}=-2.862< 0, \\ & {-}2a_{2}+a_{2}^{2}\delta_{2}^{+}+ \sum_{j=1}^{2}a_{2}\delta _{2}^{+}a_{2j}^{+}L_{jf}^{2\xi_{j}}+ \sum_{j=1}^{2}a_{2j}^{+}L_{jf}^{2\xi_{j}} \\ & \quad{}+\sum_{i=1}^{2}a_{i}^{2} \delta_{i}^{+}+\sum_{j=1}^{2}b_{2} \eta _{2}^{+}b_{2j}^{+}L_{jg}^{2(1-\varepsilon _{j})}+b_{2j}^{+}L_{jg}^{2(1-\varepsilon_{j})}=-2.04< 0, \\ & {-}2b_{1}+b_{1}^{2}\eta_{1}^{+}+ \sum_{j=1}^{2}b_{1}\eta _{1}^{+}b_{1j}^{+}L_{jg}^{2\varepsilon_{j}}+ \sum_{j=1}^{2}b_{1j}^{+}L_{jg}^{2\varepsilon_{j}} \\ & \quad{}+\sum_{i=1}^{2}a_{1}^{2} \delta_{1}^{+}a_{1j}^{+}L_{jf}^{2(1-\xi _{j})}+a_{1j}^{+}L_{jf}^{2(1-\xi_{j})}+b_{1} \eta_{1}^{+}=-3.256< 0, \\ & {-}2b_{2}+b_{2}^{2}\eta_{2}^{+}+ \sum_{j=1}^{2}b_{2}\eta _{2}^{+}b_{2j}^{+}L_{jg}^{2\varepsilon_{j}}+ \sum_{j=1}^{2}b_{2j}^{+}L_{jg}^{2\varepsilon_{j}} \\ & \quad{}+\sum_{i=1}^{2}a_{2}^{2} \delta_{2}^{+}a_{2j}^{+}L_{jf}^{2(1-\xi _{j})}+a_{2j}^{+}L_{jf}^{2(1-\xi_{j})}+b_{2} \eta_{2}^{+}=-3.024< 0. \end{aligned}$$

**Figure 1:**
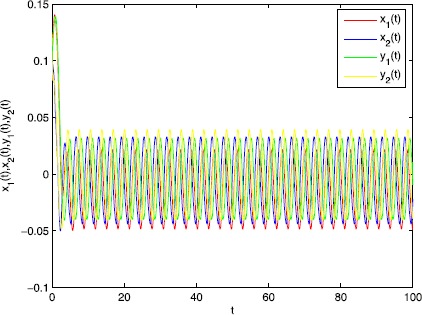
Transient response of state variables $x_{1}(t),x_{2}(t),y_{1}(t)$, and $y_{2}(t)$

Then all the conditions (H1)–(H4) hold. Thus system (4.1) has exactly one *π*-anti-periodic solution which is globally exponentially stable. The results are illustrated in Fig. 1.

## Conclusions

In this paper, we have investigated the asymptotic behavior of BAM neural networks with time-varying delays in the leakage terms. Applying the fundamental solution matrix of coefficient matrix, we obtained a series of new sufficient conditions to guarantee the existence and global exponential stability of an anti-periodic solution for the BAM neural networks with time-varying delays in the leakage terms. The obtained conditions are easy to check in practice. Finally, an example is included to illustrate the feasibility and effectiveness.

## References

[1] Guo S.J., Huang L.H. (2003). Hopf bifurcating periodic orbits in a ring of neurons with delays. Phys. D: Nonlinear Phenom..

[2] Song Y.Y., Han Y.Y., Peng Y.H. (2013). Stability and Hopf bifurcation in an unidirectional ring of n neurons with distributed delays. Neurocomputing.

[3] Ho D.W.C., Liang J.L., Lam J. (2006). Global exponential stability of impulsive high-order BAM neural networks with time-varying delays. Neural Netw..

[4] Zhang Z.Q., Yang Y., Huang Y.S. (2011). Global exponential stability of interval general BAM neural networks with reaction-diffusion terms and multiple time-varying delays. Neural Netw..

[5] Zhang Z.Q., Liu K.Y. (2011). Existence and global exponential stability of a periodic solution to interval general bidirectional associative memory (BAM) neural networks with multiple delays on time scales. Neural Netw..

[6] Zhang Z.Q., Liu W.B., Zhou D.M. (2012). Global asymptotic stability to a generalized Cohen–Grossberg BAM neural networks of neutral type delays. Neural Netw..

[7] Samidurai R., Sakthivel R., Anthoni S.M. (2009). Global asymptotic stability of BAM neural networks with mixed delays and impulses. Appl. Math. Comput..

[8] Raja R., Anthoni S.M. (2011). Global exponential stability of BAM neural networks with time-varying delays: the discrete-time case. Commun. Nonlinear Sci. Numer. Simul..

[9] Zhang Z.Q., Zhou D.M. (2010). Existence and global exponential stability of a periodic solution for a discrete-time interval general BAM neural networks. J. Franklin Inst..

[10] Lakshmanan S., Park J.H., Lee T.H., Jung H.Y., Rakkiyappan R. (2013). Stability criteria for BAM neural networks with leakage delays and probabilistic time-varying delays. Appl. Math. Comput..

[11] Li Y.K., Wang C. (2013). Existence and global exponential stability of equilibrium for discrete-time fuzzy BAM neural networks with variable delays and impulses. Fuzzy Sets Syst..

[12] Chen A.P., Huang L.H., Cao J.D. (2003). Existence and stability of almost periodic solution for BAM neural networks with delays. Appl. Math. Comput..

[13] Zhang Z.Q., Liu K.Y., Yang Y. (2012). New LMI-based condition on global asymptotic stability concerning BAM neural networks of neutral type. Neurocomputing.

[14] Zhang C.R., Zheng B.D., Wang L.C. (2009). Multiple Hopf bifurcations of symmetric BAM neural network model with delay. Appl. Math. Lett..

[15] Song Q.K., Wang Z.D. (2007). An analysis on existence and global exponential stability of periodic solutions for BAM neural networks with time-varying delays. Nonlinear Anal., Real World Appl..

[16] Sakthivel R., Arunkumar A., Mathiyalagan K., Marshal Anthoni S. (2011). Robust passivity analysis of fuzzy Cohen–Grossberg BAM neural networks with time-varying delays. Appl. Math. Comput..

[17] Jiang H.J., Teng Z.D. (2009). Boundedness, periodic solutions and global stability for cellular neural networks with variable coefficients and infinite delays. Neurocomputing.

[18] Balasubramaniam P., Vembarasan V., Rakkiyappan R. (2012). Global robust asymptotic stability analysis of uncertain switched Hopfield neural networks with time delay in the leakage term. Neural Comput. Appl..

[19] Samidurai R., Sakthivel R., Anthoni S.M. (2009). Global asymptotic stability of BAM neural networks mixed delays and impulses. Appl. Math. Comput..

[20] Li Y.K., Chen X.R., Zhao L. (2009). Stability and existence of periodic solutions to delayed Cohen–Grossberg BAM neural networks with impulses on time scales. Neurocomputing.

[21] Liu Y.G., Huang Z.X., Chen L.P. (2012). Almost periodic solution of impulsive Hopfield neural networks with finite distributed delays. Neural Comput. Appl..

[22] Balasubramaniam P., Rakkiyappan R., Sathy R. (2011). Delay dependent stability results for fuzzy BAM neural networks with Markovian jumping parameters. Expert Syst. Appl..

[23] Stamova I.M., Ilarionov R., Krustev K. (2011). Asymptotic behavior of equilibriums of a class of impulsive bidirectional associative memory neural networks with time-varying delays. Neural Comput. Appl..

[24] Park J.H., Park C.H., Kwon O.M., Lee S.M. (2008). A new stability criterion for bidirectional associative memory neural networks of neutral-type. Appl. Math. Comput..

[25] Park J.H., Lee S.M., Kwon O.M. (2009). On exponential stability of bidirectional associative memory neural networks with time-varying delays. Chaos Solitons Fractals.

[26] Balasubramaniam P., Vembarasan V. (2011). Asymptotic stability of BAM neural networks of neutral-type with impulsive effects and time delay in the leakage term. Int. J. Comput. Math..

[27] Balasubramaniam P., Vembarasan V. (2011). Robust stability of uncertain fuzzy BAM neural networks of neutral-type Markovian jumping parameters and impulses. Comput. Math. Appl..

[28] Domoshnitsky A., Sheina M.V. (1989). Nonnegativity of Cauchy matrix and stability of systems with delay. Differ. Uravn..

[29] Bainov D., Domoshnitsky A. (1993). Nonnegativity of the Cauchy matrix and exponential stability of a neutral type system of functional-differential equations. Extr. Math..

[30] Gyori I., Hartung F. (2006). Fundamental solution and asymptotic stability of linear delay differential equations. Dyn. Contin. Discrete Impuls. Syst..

[31] Berezansky L., Braverman E., Domoshnitsky A. (2011). On nonoscillation of systems of delay equations. Funkc. Ekvacioj.

[32] Agarwal R.P., Berezansky L., Braverman E., Domoshnitsky A. (2012). Nonoscillation Theory of Functional Differential Equations with Applications.

[33] Domoshnitsky A., Gitman M., Shklyar R. (2014). Stability and estimate of solution to uncertain neutral delay systems. Bound. Value Probl..

[34] Domoshnitsky A., Fridman E. (2016). A positivity-based approach to delay-dependent stability of systems with large time-varying delays. Syst. Control Lett..

[35] Tang X.H., Chen S.T. (2017). Ground state solutions of Nehari–Pohozaev type for Kirchhoff-type problems with general potentials. Calc. Var. Partial Differ. Equ..

[36] Chen S.T., Tang X.H. (2016). Ground state sign-changing solutions for a class of Schrödinger–Poisson type problems in $R^{3}$. Z. Angew. Math. Phys..

[37] Tang X.H., Chen S.T. (2017). Ground state solutions of Nehari–Pohozaev type for Schrödinger–Poisson problems with general potentials. Discrete Contin. Dyn. Syst., Ser. A.

[38] Xu C.J., Li P.L. (2017). Global exponential stability of periodic solution for fuzzy cellular neural networks with distributed delays and variable coefficients. J. Intell. Fuzzy Syst..

[39] Xu C.J., Li P.L. (2017). *p*th moment exponential stability of stochastic fuzzy Cohen–Grossberg neural networks with discrete and distributed delays. Nonlinear Anal., Model. Control.

[40] Xu C.J., Li P.L. (2018). Global exponential convergence of fuzzy cellular neural networks with leakage delays, distributed delays and proportional delays. Circuits Syst. Signal Process..

[41] Xu C.J., Li P.L. (2018). On anti-periodic solutions for neutral shunting inhibitory cellular neural networks with time-varying delays and D operator. Neurocomputing.

[42] Kosko B. (1987). Adaptive bi-directional associative memories. Appl. Opt..

[43] Haykin S. (1994). Neural Networks.

[44] Kosok B. (1992). Neural Networks and Fuzzy Systems.

[45] Gopalsamy K. (1992). Stability and Oscillations in Delay Differential Equations of Populations Dynamics.

[46] Rakkiyappan R., Chandrasekar A., Lakshmanan S., Park J.H., Jung H.Y. (2013). Effects of leakage time-varying delays in Markovian jump neural networks with impulse control. Neurocomputing.

[47] Balasubramaniam P., Vembarasan V., Rakkiyappan R. (2011). Leakage delays in T-S fuzzy cellular neural networks. Neural Process. Lett..

[48] Gopalsamy K. (2007). Leakage terms in BAM. J. Math. Anal. Appl..

[49] Liu B.W. (2013). Global exponential stability for BAM neural networks with time-varying delays in the leakage terms. Nonlinear Anal., Real World Appl..

[50] Wei X.R., Qiu Z.P. (2013). Anti-periodic solutions for BAM neural networks with time delays. Appl. Math. Comput..

[51] Liu D.Y., Wu W.J., Liu H.T., Zhang J.W. (2011). Anti-periodic solutions for interval general bidirectional associative memory (BAM) neural networks with impulses on time scales. J. Inf. Comput. Sci..

[52] Li Y.K., Yang L. (2009). Anti-periodic solutions for Cohen–Grossberg neural networks with bounded and unbounded delays. Commun. Nonlinear Sci. Numer. Simul..

[53] Shao J.Y. (2008). Anti-periodic solutions for shunting inhibitory cellular neural networks with time-varying delays. Phys. Lett. A.

[54] Fan Q.Y., Wang W.T., Yi X.J. (2009). Anti-periodic solutions for a class of nonlinear *n*th-order differential equations with delays. J. Comput. Appl. Math..

[55] Li Y.K., Xu E.L., Zhang T.W. (2010). Existence and stability of anti-periodic solution for a class of generalized neural networks with impulsives and arbitrary delays on time scales. J. Inequal. Appl..

[56] Gong S.H. (2009). Anti-periodic solutions for a class of Cohen–Grossberg neural networks. Comput. Math. Appl..

[57] Ou C.X. (2008). Anti-periodic solutions for high-order Hopfield neural networks. Comput. Math. Appl..

[58] Peng G.Q., Huang L.H. (2009). Anti-periodic solutions for shunting inhibitory cellular neural networks with continuously distributed delays. Nonlinear Anal., Real World Appl..

[59] Huang Z.D., Peng L.Q., Xu M. (2010). Anti-periodic solutions for high-order cellular neural networks with time-varying delays. Electron. J. Differ. Equ..

[60] Zhang A.P. (2013). Existence and exponential stability of anti-periodic solutions for HCNNs with time-varying leakage delays. Adv. Differ. Equ..

[61] Li Y.K., Yang L., Wu W.Q. (2011). Anti-periodic solutions for a class of Cohen–Grossberg neural networks with time-varying on time scales. Int. J. Syst. Sci..

[62] Pan L.J., Cao J.D. (2011). Anti-periodic solution for delayed cellular neural networks with impulsive effects. Nonlinear Anal., Real World Appl..

[63] Li Y.K. (2011). Anti-periodic solutions to impulsive shunting inhibitory cellular neural networks with distributed delays on time scales. Commun. Nonlinear Sci. Numer. Simul..

[64] Peng L., Wang W.T. (2013). Anti-periodic solutions for shunting inhibitory cellular neural networks with time-varying delays in leakage terms. Neurocomputing.

[65] Shi P.L., Dong L.Z. (2010). Existence and exponential stability of anti-periodic solutions of Hopfield neural networks with impulses. Appl. Math. Comput..

